# Supplementary material for: Disproportionately Elevated Sulcal Index (DESI): An automatically driven index representing disproportionate subarachnoid space enlargement in brain MRI scans
Source: medRxiv. 2025 Dec 2:2025.12.01.25341388. Preprint. [Version 1] doi: 10.64898/2025.12.01.25341388 (PMC12704640; doi:10.64898/2025.12.01.25341388)
Supplement: Supplement 1 [file media-1.docx]

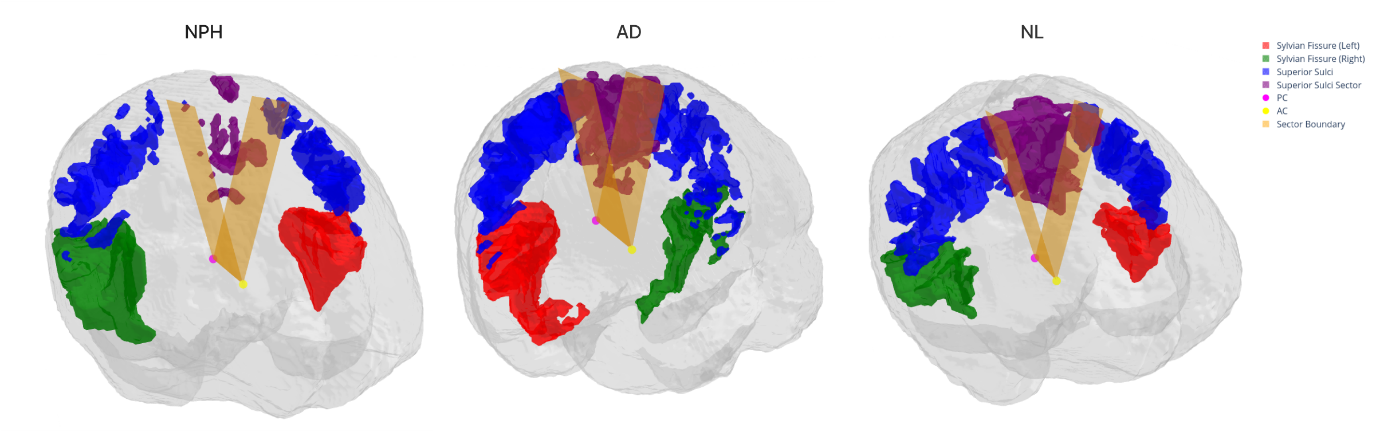


Supplementary Figure 1. Three-dimensional renderings of segmented Sylvian fissures and suprasylvian sulcal spaces within the 30° sector defined by the AC-PC line, shown for a cognitively normal individual, a patient with Alzheimer’s disease, and a NPH patient with DESH.
